# Synaptopathy in the TDP‐43ΔNLS Mouse Model of Sporadic Amyotrophic Lateral Sclerosis

**DOI:** 10.1111/ejn.70320

**Published:** 2025-11-17

**Authors:** Ani Ayvazian‐Hancock, Emma Butler, Claire F. Meehan, Gareth B. Miles, Matthew J. Broadhead

**Affiliations:** ^1^ School of Psychology & Neuroscience University of St Andrews St Andrews UK; ^2^ University of Copenhagen Copenhagen Denmark; ^3^ Centre of Biophotonics University of St Andrews St Andrews UK

## Abstract

Sporadic cases of amyotrophic lateral sclerosis (sALS) represent the most common form of motor neuron disease. sALS is characterised by pathological cytoplasmic inclusions of TDP‐43, so‐called reactive astrocyte pathology and motor neuron degeneration. Alterations in certain subpopulations of synapses between neurons are thought to be a key driver of the pathological mechanisms of ALS. However, we do not have a clear understanding of which types of synapses are impacted in ALS. Identifying vulnerable synapses affected in sALS models may provide insights into the key sites of disease pathogenesis. In this study we have performed quantitative high‐resolution microscopy to survey different synapse subtypes, including excitatory (glutamatergic), inhibitory (glycinergic) and modulatory (cholinergic C‐Boutons) synapses, in the spinal cord of a mouse model of sALS showing inducible TDP‐43 pathology (TDP43ΔNLS) restricted to neurons. We have identified changes in cholinergic synapses and a subpopulation of excitatory synapses. Mice display robust neuronal TDP‐43 pathology and evidence of TDP‐43 changes at cholinergic C‐boutons. We also observe no evidence of astrocytic pathology nor changes in the fraction of synapses that are contacted by astrocytes. Overall, our findings highlight the selective vulnerability of distinct synapse populations in ALS.

## Graphical abstract

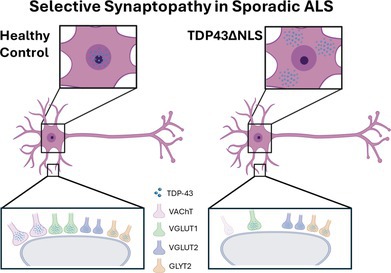

**Abbreviations**
ALS: amyotrophic lateral sclerosis
C9ORF72: chromosome 9 open reading frame 72
Camk2a: calcium/calmodulin‐dependent protein kinase II‐alpha
Dox: doxycycline
fALS: familial ALS
FUS: RNA‐binding protein fused in sarcoma
GFAP: glial fibrillary acidic protein
GLYT2: glycine transporter 2
MN: motor neuron
NEFH: neurofilament heavy
PCR: polymerase chain reaction
PSD95: postsynaptic density protein‐95
pTDP‐43: phosphorylated TDP‐43
sALS: sporadic ALS
SOD1: Cu/Zn superoxide dismutase 1
TDP43: TAR DNA Binding Protein 43
VAChT: vesicular acetylcholine transporter
VGLUT1: vesicular glutamate transporter 1
VGLUT2: vesicular glutamate transporter 2
ΔNLS: nuclear localization signal

## 1 Introduction

Amyotrophic lateral sclerosis (ALS) is a neurodegenerative disorder characterised by motor neuron (MN) cell death in the nervous system, which leads to behavioural symptoms including muscle weakening, and eventually death due to paralysis of respiratory muscles (Ragagnin et al. 2019; Brown and Al‐Chalabi 2017). ALS may be caused by a combination of genetic mutations or environmental risk factors (Zarei et al. 2015). Whilst a small proportion of ALS cases are inherited and classified as familial ALS (fALS), approximately 90% of patients have sporadic ALS (sALS), with no known family history of the disease (Millecamps et al. 2010; Picher‐Martel et al. 2016; McCann et al. 2023). Over 30 different gene mutations are implicated in the aetiology of ALS, including mutations in genes encoding guanine nucleotide exchange chromosome 9 open reading frame 72 (C9ORF72), Cu/Zn superoxide dismutase 1 (SOD1) RNA‐binding protein Fused in Sarcoma (FUS) and TAR DNA‐binding protein 43 (TDP43).

Despite the complex and diverse genetic and molecular aetiology, approximately 97% of ALS cases display TDP‐43 pathology, characterised by the mislocalisation and aggregation of hyper‐phosphorylated TDP‐43 (pTDP‐43) inclusions in the cytoplasm of neurons (Mackenzie et al. 2010; Mackenzie 2007; Trist et al. 2022; Chen and Cohen 2019; McGurk et al. 2020). TDP‐43 has many cellular functions including RNA splicing and translation (Ayala et al. 2008; Tollervey et al. 2011; Cohen et al. 2011). Whilst TDP‐43 is predominantly expressed in the nucleus, it is also expressed in lower concentrations throughout the cell cytoplasm, including at presynaptic and postsynaptic compartments (Wang et al. 2008; Broadhead et al. 2023), where it may have a role in local translational processes related to synaptic proteins (Tollervey et al. 2011; Godena et al. 2011; Sephton et al. 2011; Colombrita et al. 2012). TDP‐43 pathology may represent a conserved mechanism driving neurodegeneration across the diverse spectrum of ALS patients.

Before the loss of MNs in the spinal cord, central chemical synapses between neurons are highly susceptible to structural and functional changes that may drive early‐stage hyper‐excitability in MNs, which is hypothesised to lead to excitotoxicity and subsequent MN cell death (Fogarty 2019). MNs are the final common pathway of the nervous system, receiving a wide range of different synaptic inputs. Synapses are highly diverse in their molecular composition, morphology and function, both within and between different neural circuits and anatomical regions of the nervous system (Nagy et al. 1993; Goshgarian and Rafols 1984; Witts et al. 2014; Broadhead et al. 2020; Todd et al. 2003). Synaptic diversity may also incur selective vulnerability of molecularly or structurally distinct synapse subtypes (Grant et al. 2016; Grant and Fransén 2020). For example, tripartite synapses have previously been shown to be selectively vulnerable to degeneration in ALS compared to synapses not contacted by astrocytes (Broadhead et al. 2022). A range of other studies indicate changes in other classes of synapses in ALS, including excitatory (Bączyk et al. 2020; Fogarty et al. 2016), inhibitory (Montañana‐Rosell et al. 2024; Foerster et al. 2013; Nascimento et al. 2024) and cholinergic synapses (Herron and Miles 2012; Wells et al. 2021) in the spinal cord. Therefore, identifying which types of synapses are most significantly impacted in ALS is critical for understanding the mechanisms of the disease and may help identify critical pathways for targeted therapeutic strategies.

In this study, we examined different types of synapses in the lateral motor pools of the upper‐lumbar spinal cord (Lumbar Segments 2–3, L2–L3) in a mouse model of sALS. This model of sALS, first demonstrated in 2015 (Walker et al. 2015), has doxycycline (Dox)‐suppressible expression of human TDP‐43 (hTDP‐43) harbouring a defective nuclear localization signal (∆NLS) under the control of the neurofilament heavy chain promoter (NEFH). The model shows TDP‐43 mislocalisation to the soma of brain and spinal neurons and resultant motor deterioration from 2 to 4 weeks post‐induction and a significant decline in mortality by 6–10 weeks (Walker et al. 2015; Bak et al. 2023; Djukic et al. 2025). Using immunohistochemistry and Airyscan confocal microscopy, we identified selective vulnerability of certain synapse subtypes in symptomatic‐stage 4‐week induced animals compared to controls. Strikingly, however, we revealed a strong concordance in the hallmarks of synaptic pathology in subtypes of excitatory synapses between this TDP‐43–associated model and other models harbouring different genetic mutations. These findings illustrate conserved hallmarks of synaptopathy across the diverse spectrum of ALS that may point towards a convergent mechanism.

## 2 Methods

### 2.1 Ethics

All procedures on live animals were approved by the Danish Animal Experiments Inspectorate (Permission number 2018‐15‐0201‐01426) and were conducted in accordance with the EU Directive 2010/63/EU. Once mouse tissue was shipped to the UK, all procedures were conducted in accordance with the Scientific Procedures UK Animals Act 1986.

### 2.2 TDP43ΔNLS Mouse Model and Behavioural Assessment

Mice were produced by crossing a tetO‐hTDP‐43‐ΔNLS line 4 (https://www.jax.org/strain/014650) with a NEFH‐tTA line 8 (https://www.jax.org/strain/025397). This cross breeding created a mouse model with tetracycline‐repressible TDP‐43 that lacks the nuclear localization signal (ΔNLS). When the Dox is removed from the diet of bigenic animals, toxic mislocalisation of TDP‐43 into the cytoplasm of neurons is induced (Walker et al. 2015). Contrary to other versions of this mouse model that expressed defective TDP43ΔNLS under brain‐specific promoters (Igaz et al. 2011), the NEFH promoter ensures both upper and lower MN phenotypes that resemble ALS as opposed to primary lateral sclerosis which predominantly affects upper MNs. Mice were genotyped using a standard PCR assay described in a previous study. Four transgene‐specific primers were used for PCR amplification to enable the final detection of these sequences on electrophoretic gels; 5′‐CTC GCG CAC CTG CTG AAT‐3′ (Tg [NEFH‐tTA] forward), 5′‐CAG TAC AGG GTA GGC TGC TC‐3′(Tg (NEFH‐tTA) reverse), 5′‐TTG CGT GAC TCT TTA GTA TTG GTT TGA TGA‐3′ (Tg [tetO‐hTDP‐43‐ΔNLS] forward) and 5′‐CTC ATC CAT TGC TGC TGC G‐3′ (Tg [tetO‐hTDP‐43‐ΔNLS] reverse) (Bak et al. 2023; Djukic et al. 2025). In our study, all mice underwent withdrawal of Dox from the diet at the same time point (7 weeks old) for a total of 4 weeks (11 weeks old mice at the point of behavioural assessment and euthanasia). Mice expressing only the tTA gene were designated as control animals, whilst mice expressing both the NEFH‐tTA and TetO promoter (bigenic) were designated as TDP43ΔNLS animals exhibiting symptoms of sALS. At the experiment end‐stage, 4 weeks after induction began, the ALS‐like phenotype was confirmed using a simple behavioural test. In the 2‐min tail suspension test, mice are gently suspended by their tail for up to 2 min. Healthy mice will normally extend out their hind limbs during this task but mice with motor dysfunction display a distinctive hindlimb clasping behaviour. In the grip strength endurance test, mice are placed on a metal grid which is gently shaken to encourage them to grip on. It is then inverted over a soft surface and the endurance time that the mouse can hold on for is recorded. Assessment in the grip endurance test was performed for up to a maximum of 2 min to avoid evoking prolonged stress in the animals. The mean of three trials (with a 10‐min break between each trial) was calculated. The 4 weeks induction was elected for this study to ensure that the animals developed clear TDP‐43 mislocalisation and significant motor deficits, prior to severe signs of suffering such as 20% weight loss or being unable to right themselves in accordance with our ethically approved procedures.

### 2.3 Tissue Collection

**FIGURE 1:**
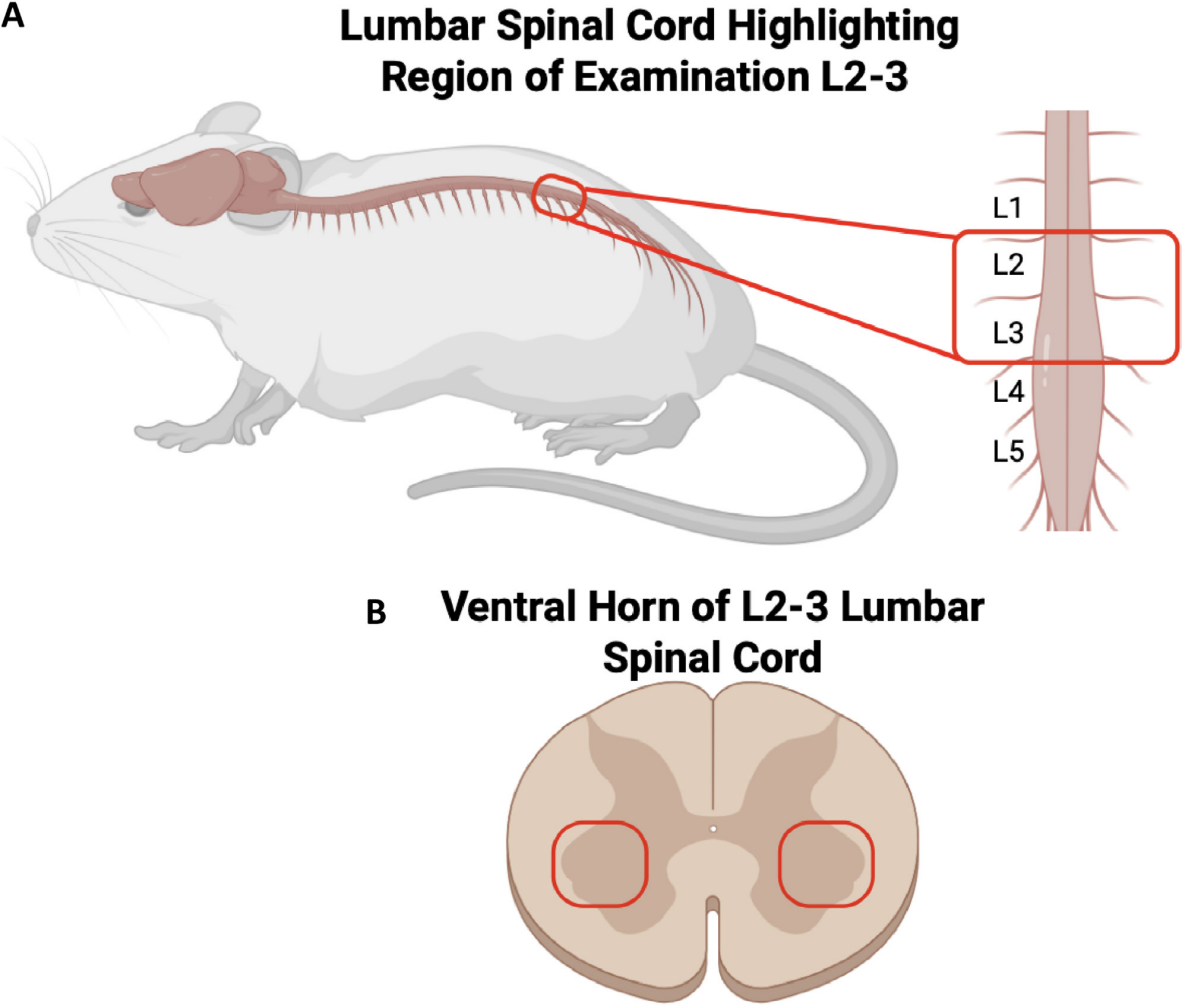
Schematic displaying lumbar spinal cord regions of interest. (A) Enlarged model of adult mouse lumbar spinal cord, highlighting sectioned regions, L2–L3. (B) Cross section of upper lumbar spinal cord, highlighting ventral horns, motorneuron assessment region. Created in BioRender. Ayvazian‐hancock, A. (2025) https://BioRender.com/k1azkxb.

Animals were euthanized and tissue was collected at 11 weeks of age (4 weeks post‐induction). Euthanasia was performed with Sodium Pentobarbital (120 mg/kg), and animals were transcardially perfused with 0.9% saline followed by 4% paraformaldehyde. Spinal cords were then removed and post‐fixed for 2 h in 4% paraformaldehyde. Spinal cords were then immersed in 30% sucrose in phosphate‐buffered saline (PBS) overnight for cryoprotection. Tissue was then cryo‐embedded in OCT compound and stored at −80°C. Cryosections, at 20 μm thickness, were obtained from the L2–L3 of the spinal cord (Figure 1), using a Leica CM1860 cryostat, mounted on Leica X‐tra adhesive slides and stored at −80°C for long‐term preservation.

### 2.4 Immunohistochemistry

**FIGURE 2:**
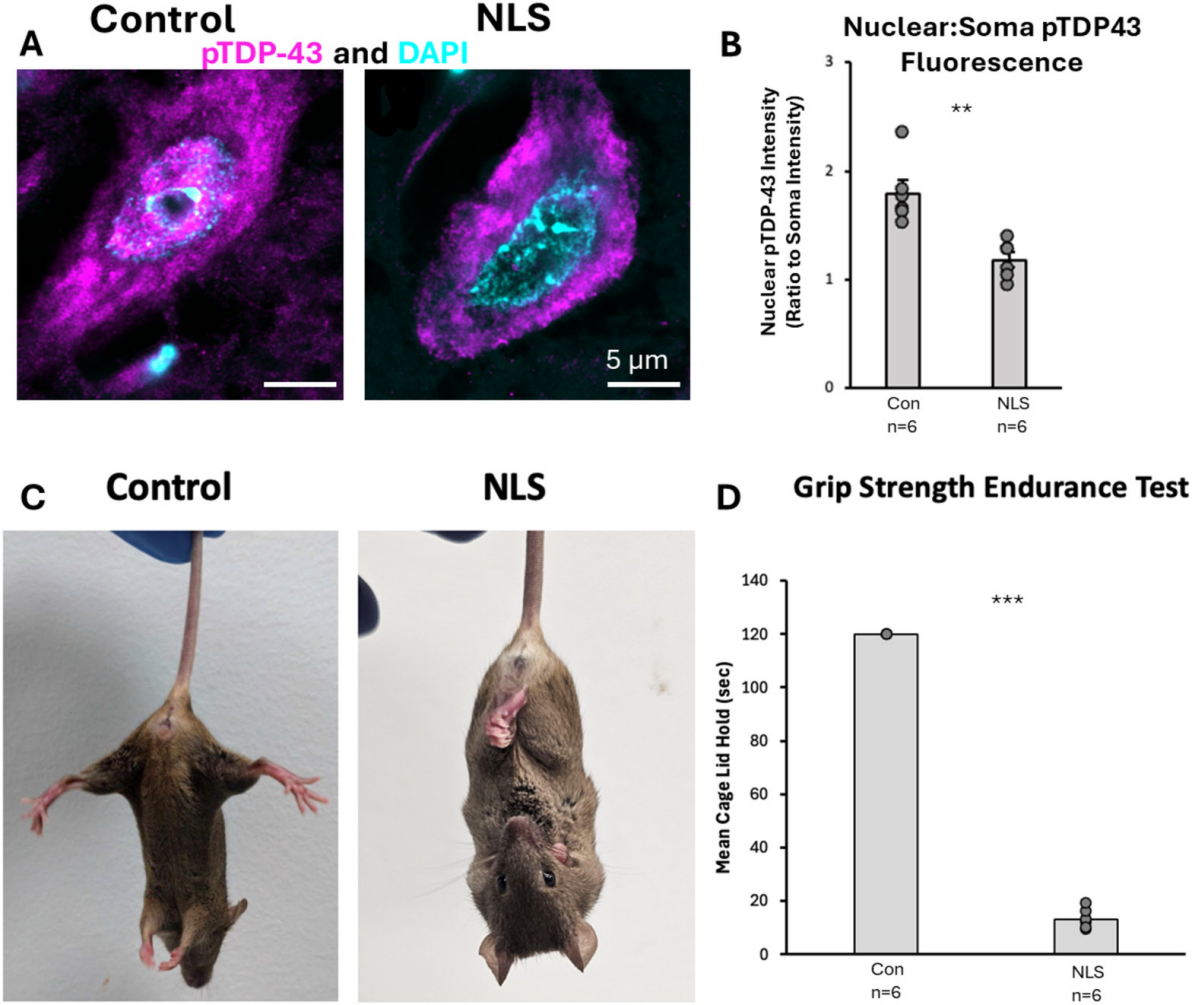
Characterisation of TDP‐43 pathology and behavioural phenotype of sALS model. (A) Immunohistochemical images staining for pTDP‐43 and DAPI in order to quantify the localisation of pTDP‐43 to the nucleus in both the TDP43ΔNLS mouse model and controls. (B) Bar graph representation of nuclear localisation of pTDP‐43 found significantly less nuclear pTDP‐43 in the TDP43ΔNLS mouse model compared to non‐transgenic controls (*t*(_10_) = 4.34, *p* = 0.002). (C) Image of non‐transgenic control mouse displaying a healthy splayed limb phenotype (left), and an image of TDP43ΔNLS mouse model displaying a severe clasping phenotype affecting all four limbs (right). (D) Two‐tailed *t*‐test bar graph representation of grip strength endurance test showed a significant decrease in mean cage lid holding time in the TDP43ΔNLS mouse model compared to non‐transgenic controls (*t*(_10_) = 71.499, *p* < 0.001).

**FIGURE 3:**
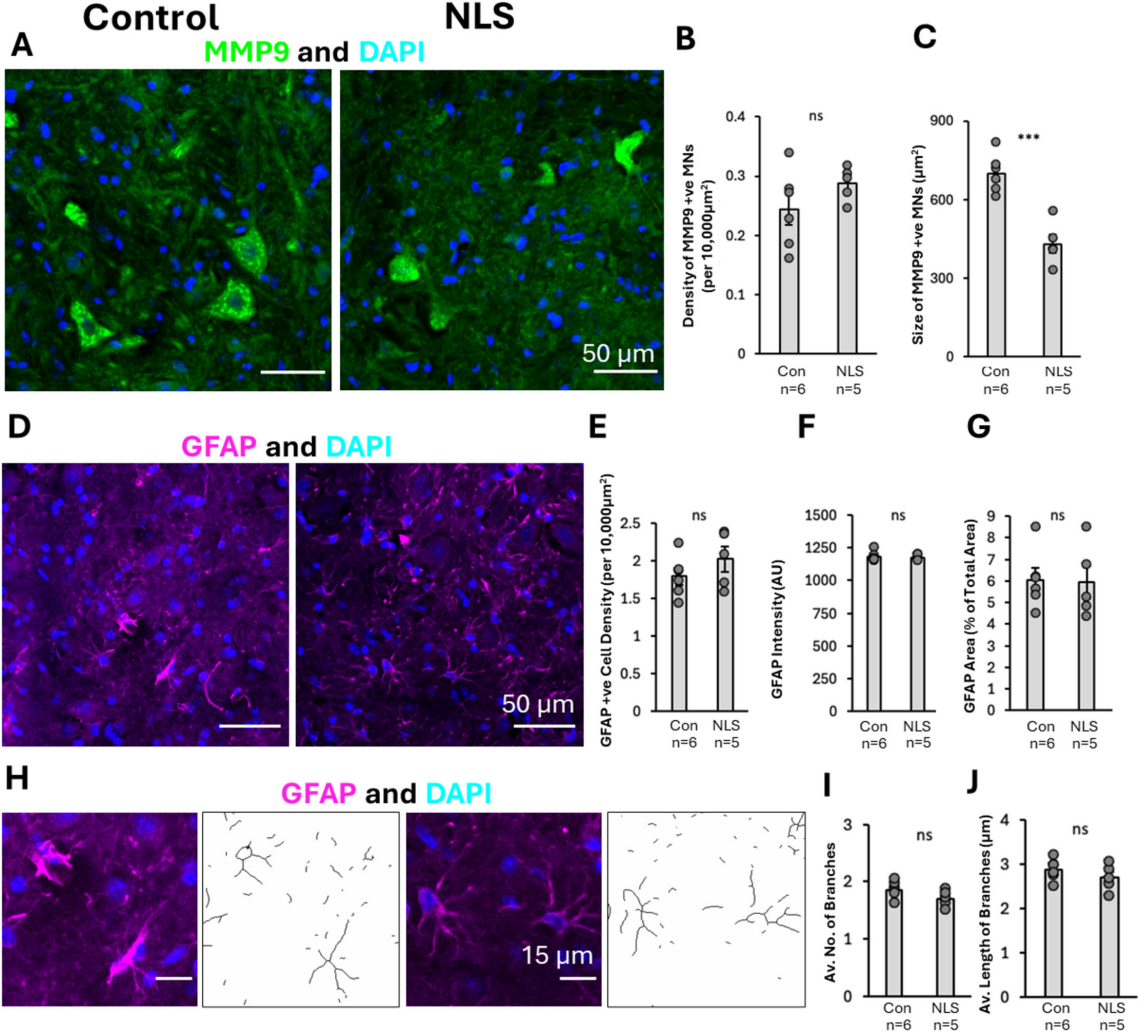
Characterising the cellular pathology in TDP43ΔNLS mouse model. (A) Immunohistochemical image assessment of MNs using MMP9 + ve and DAPI stains in order to quantify the density and size of MNs in the ventral horn of the lumbar spinal cord. (B) Two‐tailed *t*‐test bar graph representation of density of MNs showed no significant difference between the TDP43ΔNLS mouse model and non‐transgenic controls (*t*(_9_) = 1.351, *p* = 0.201). (C) Two‐tailed *t*‐test bar graph representation of size of MNs showed a significant reduction in the size of MNs in the TDP43ΔNLS mouse model when compared to controls (*t*(_9_) = 5.760, *p* < 0.001). (D) Immunohistochemical assessment of astrocytes in the TDP43ΔNLS mouse model utilising a GFAP and DAPI stain. (E) Two‐tailed *t*‐test bar graph assessment of GFAP cell density in the ventral horn of the mouse lumbar spinal cord, which showed no significant changes between the ΔNLS mouse model and controls (*t*(_9_) = 1.195, *p* = 0.262). (F) Two‐tailed *t*‐test bar graph assessment of GFAP intensity showed no significant changes between the experimental condition and controls (*t*(_9_) = 0.427, *p* = 0.679) (G). Two‐tailed *t*‐test bar graph assessment of total GFAP area showed no significant changes between ΔNLS mouse model and non‐transgenic controls (*t*(_9_) = 0.107, *p* = 0.917). (H) Immunohistochemical images and masks of GFAP to determine number and length of astrocytic branches in ΔNLS mouse model compared to controls. (I) Two‐tailed *t*‐test bar graph assessment of number of astrocytic branches showed no significant difference between ΔNLS mice when compared with controls (*t*(_9_) = 1.710, *p* = 0.121). (J) Two‐tailed *t*‐test bar graph assessment of length of astrocytic branches revealed no significant changes between the ΔNLS mouse model and controls (*t*(_9_) = 1.151, *p* = 0.279).

**FIGURE 4:**
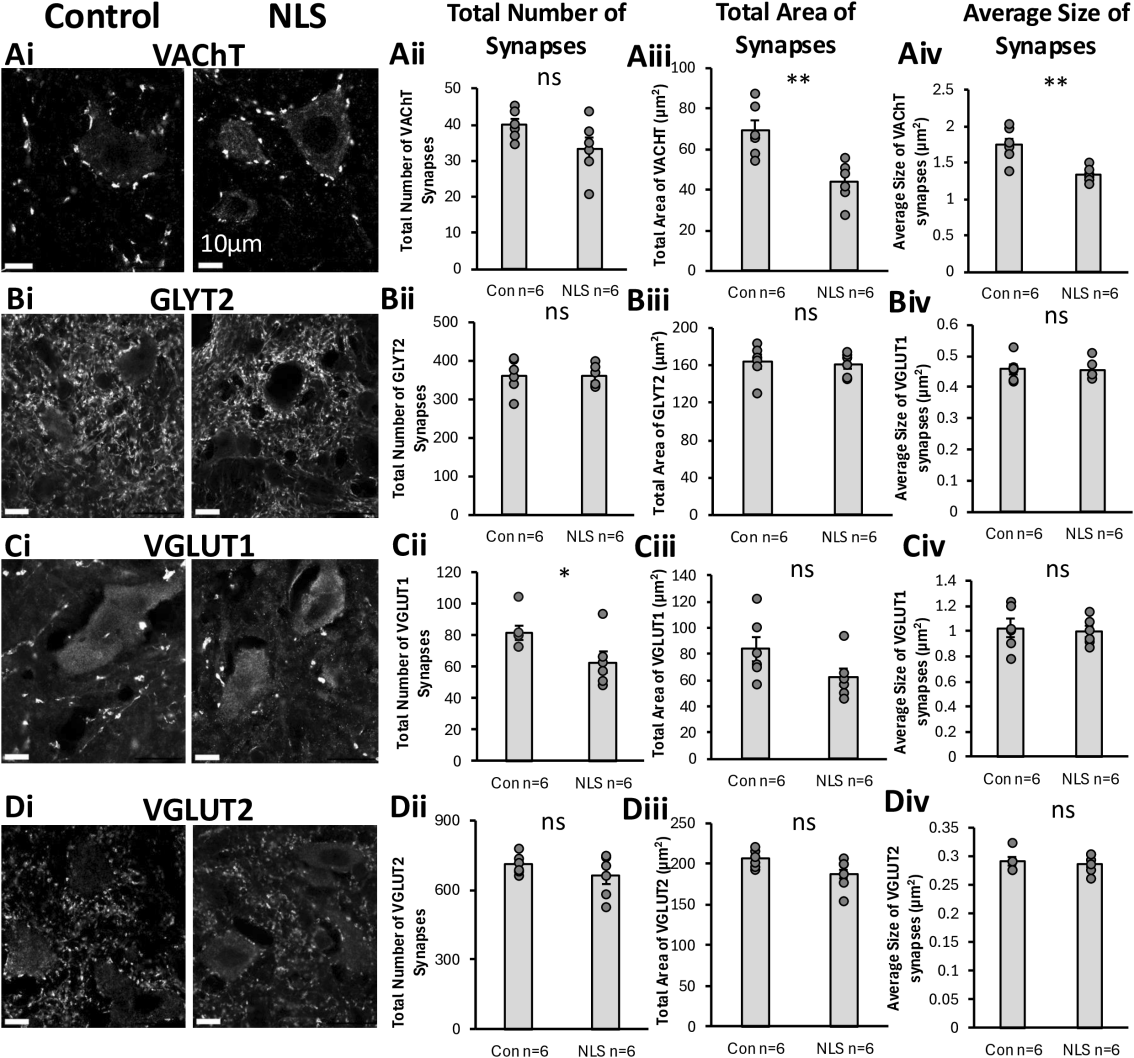
VAChT, GLYT2, VGUT1 and VGLUT2 general synaptic assessment. (Ai) Immunohistochemical staining of VAChT synapses. (Aii–Aiv) Bar graph representation of total number, area and size of VAChT synapses showing no significant changes in the number of VAChT synapses (*t*(_10_) = 1.848, *p* = 0.094), but a significant reduction in the total area coverage (*t*(_10_) = 3.805, *p* = 0.003) and average size of VAChT synapses (*t*(_10_) = 3.932, *p* = 0.003) in the TDP43ΔNLS mice compared to controls. (Bi) Immunohistochemical staining of GLYT2 synapses. (Bii–Biv) Bar graph representation of total number (*t*(_10_) = 0.104, *p* = 0.919), area (*t*(_10_) = 0.265, *p* = 0.796) and size (*t*(_10_) = 0.146, *p* = 0.444) of GLYT2 synapses showing no significant changes between TDP43ΔNLS mice and controls. (Ci) Immunohistochemical staining of VGLUT1 synapses. (Cii–Civ) Bar graph representation of total number, area and size of VGLUT1 synapses showed a significant reduction in the number of VLGUT1 synapses in the TDP43ΔNLS mouse model compared to controls (*t*(_10_) = 2.278, *p* = 0.046), but no differences in the total area coverage (*t*(_10_) = 1.793, *p* = 0.103) or average size (*t*(_10_) = 0.364, *p* = 0.723) of VGLUT1 synapses between conditions. (Di) Immunohistochemical staining of VGLUT2 synapses. (Dii–Div) Bar graph representation of total number (*t*(_10_) = 1.259, *p* = 0.237), area (*t*(_10_) = 2.059, *p* = 0.066) and size (*t*(_10_) = 0.417, *p* = 0.685) of GLYT2 synapses showing no significant changes between TDP43ΔNLS mice and controls.

**FIGURE 5:**
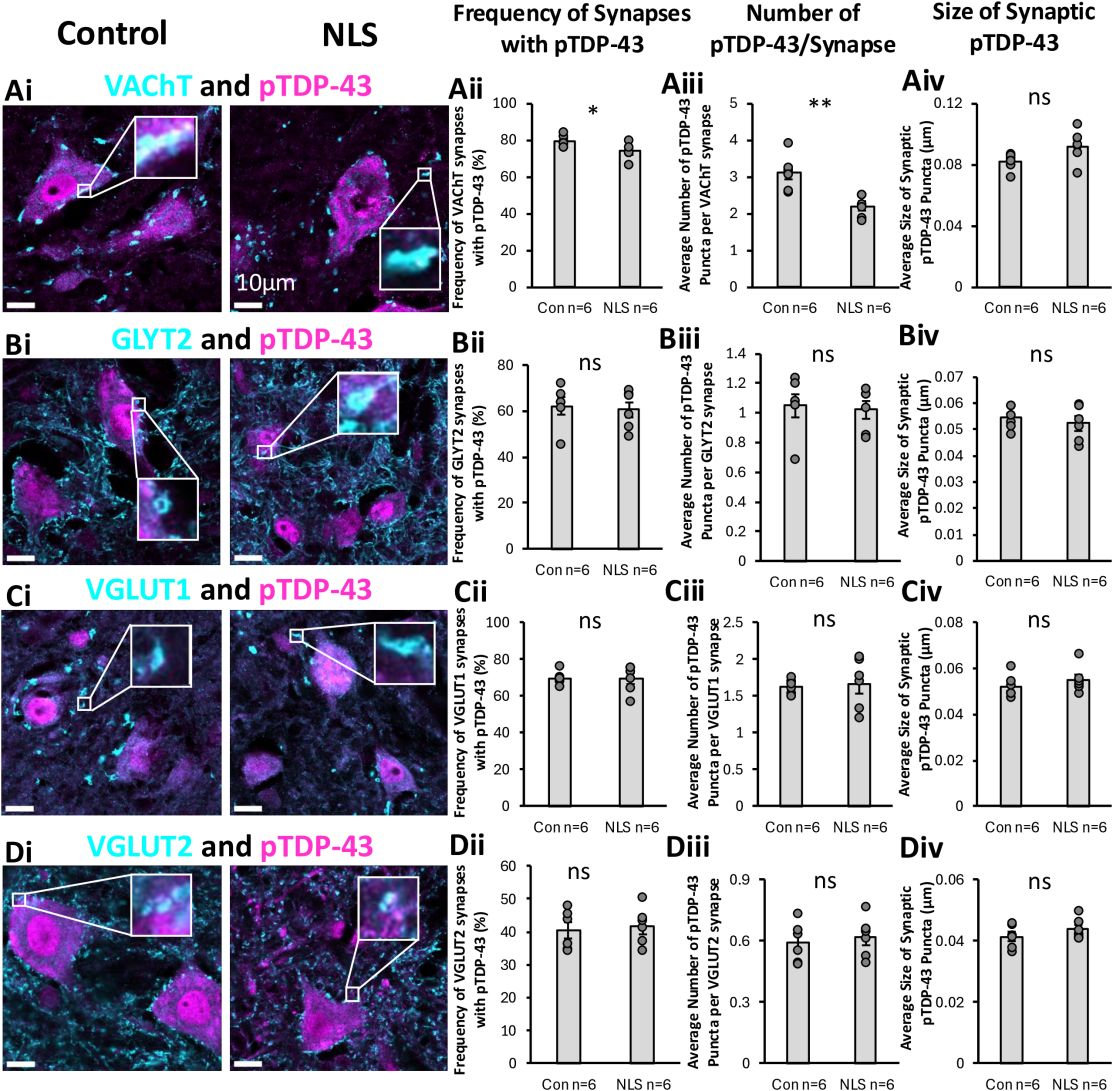
Assessment of pTDP‐43 associated with VAChT, GLYT2, VGLUT1 and VGLUT2 synapses. (Ai) Immunohistochemical stain of VAChT and pTDP‐43 to assess synaptically colocalized pTDP‐43 expression. (Aii–Aiv) Bar graph representation of pTDP‐43 colocalized to VAChT showing a significantly reduced frequency of VAChT synapses colocalized to VAChT (*t*(_10_) = 2.405, *p* = 0.037), a significantly reduced number of pTDP‐43 puncta colocalized per VAChT synapse (*t*(_10_) = 3.946, *p* = 0.003) and no change in the size of pTDP‐43 colocalized to VAChT synapses (*t*(_10_) = 2.020, *p* = 0.071), in TDP43ΔNLS mice compared to controls. (Bi) Immunohistochemical stain of GLYT2 and pTDP‐43 to assess synaptically colocalized pTDP‐43 expression. (Bii–Biv) Bar graph representations showing no changes in frequency (*t*(_10_) = 0.318, *p* = 0.757), puncta per synapse (*t*(_10_) = 0.280, *p* = 0.785) or size of pTDP‐43 colocalized with GLYT2 synapses (*t*(_10_) = 0.651, *p* = 0.529), between experimental conditions. (Ci) Immunohistochemical stain of GLYT2 and pTDP‐43 to assess synaptically colocalized pTDP‐43 expression. (Cii–Civ) Bar graph representations showing no changes in frequency (*t*(_10_) = 0.147, *p* = 0.886), puncta per synapse (*t*(_10_) = 0.320, *p* = 0.756) or size of pTDP‐43 colocalized with VGLUT1 synapses (*t*(_10_) = 0.966, *p* = 0.357), between experimental conditions. (Di) Immunohistochemical stain of GLYT2 and pTDP‐43 to assess synaptically colocalized pTDP‐43 expression. (Dii–Div) Bar graph representations showing no changes in frequency (*t*(_10_) = 0.398, *p* = 0.699), puncta per synapse (*t*(_10_) = 0.448, *p* = 0.664) or size of pTDP‐43 colocalized with VGLUT2 synapses (*t*(_10_) = 1.225, *p* = 0.249), between experimental conditions.

**FIGURE 6:**
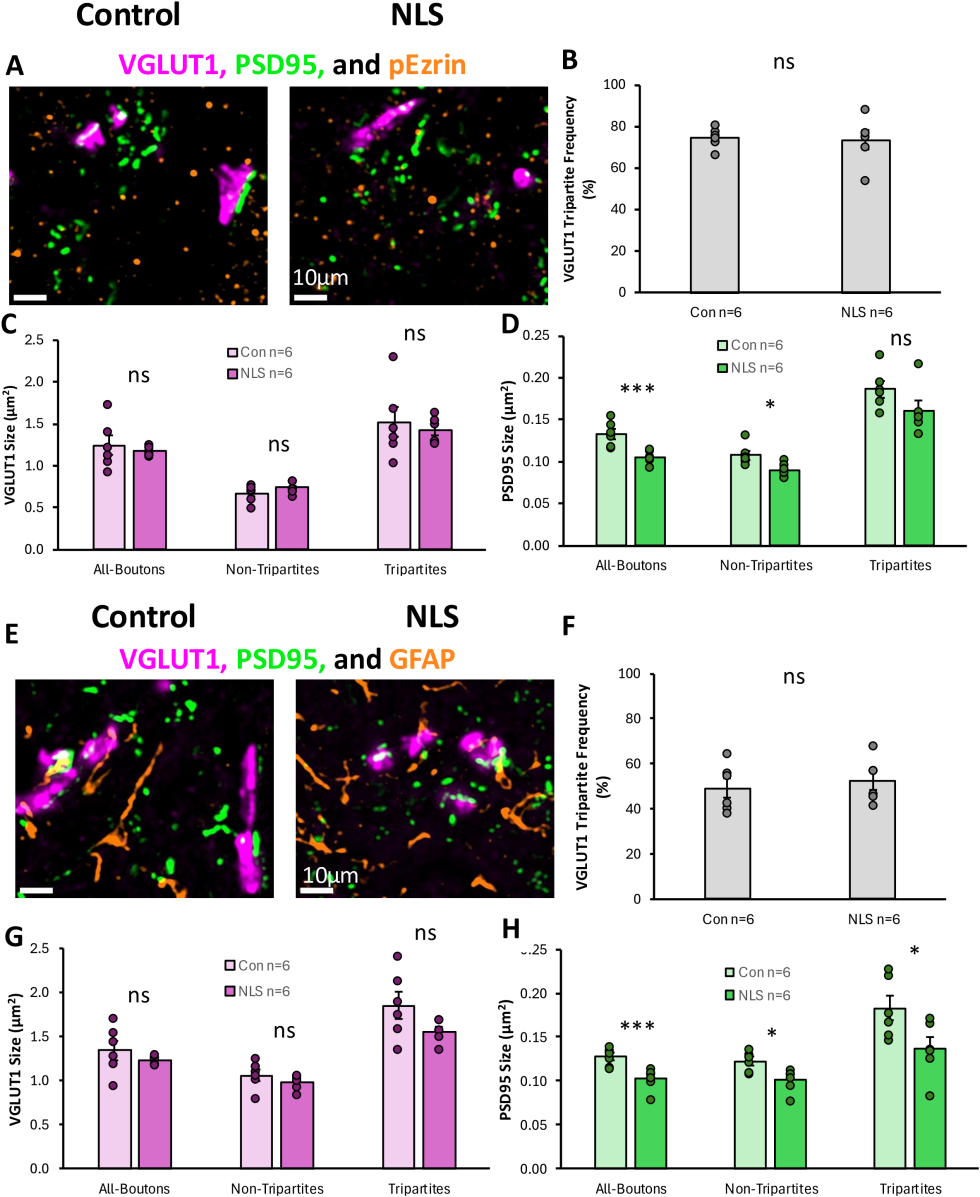
Excitatory VGLUT1‐associated Tripartite Synapses in sALS. (A) Immunohistochemical stain of VGLUT1, PSD95 and pEzrin to identify excitatory tripartite synapses. (B) Bar graph plotting the percentage of VGLUT1 synapses that are tripartite (pEzrin contacted) in control and TDP43ΔNLS (NLS) mice. (C) Bar graph of the size of VGLUT1 boutons associated with all synapses, tripartite and non‐tripartite synapses, in control and NLS mice. (D) Bar graph of the size of PSDs associated with all VGLUT1 synapses, tripartite and non‐tripartite synapses in control and NLS mice. (E) Immunohistochemical stain of VGLUT1, PSD95 and GFAP to identify excitatory tripartite synapses. (F) Bar graph plotting the percentage of VGLUT1 synapses that are tripartite (GFAP contacted) in control and TDP43ΔNLS (NLS) mice. (G) Bar graph of the size of VGLUT1 boutons associated with all synapses, tripartite and non‐tripartite synapses, in control and NLS mice. (H) Bar graph of the size of PSDs associated with all VGLUT1 synapses, tripartite and non‐tripartite synapses in control and NLS mice.

**FIGURE 7:**
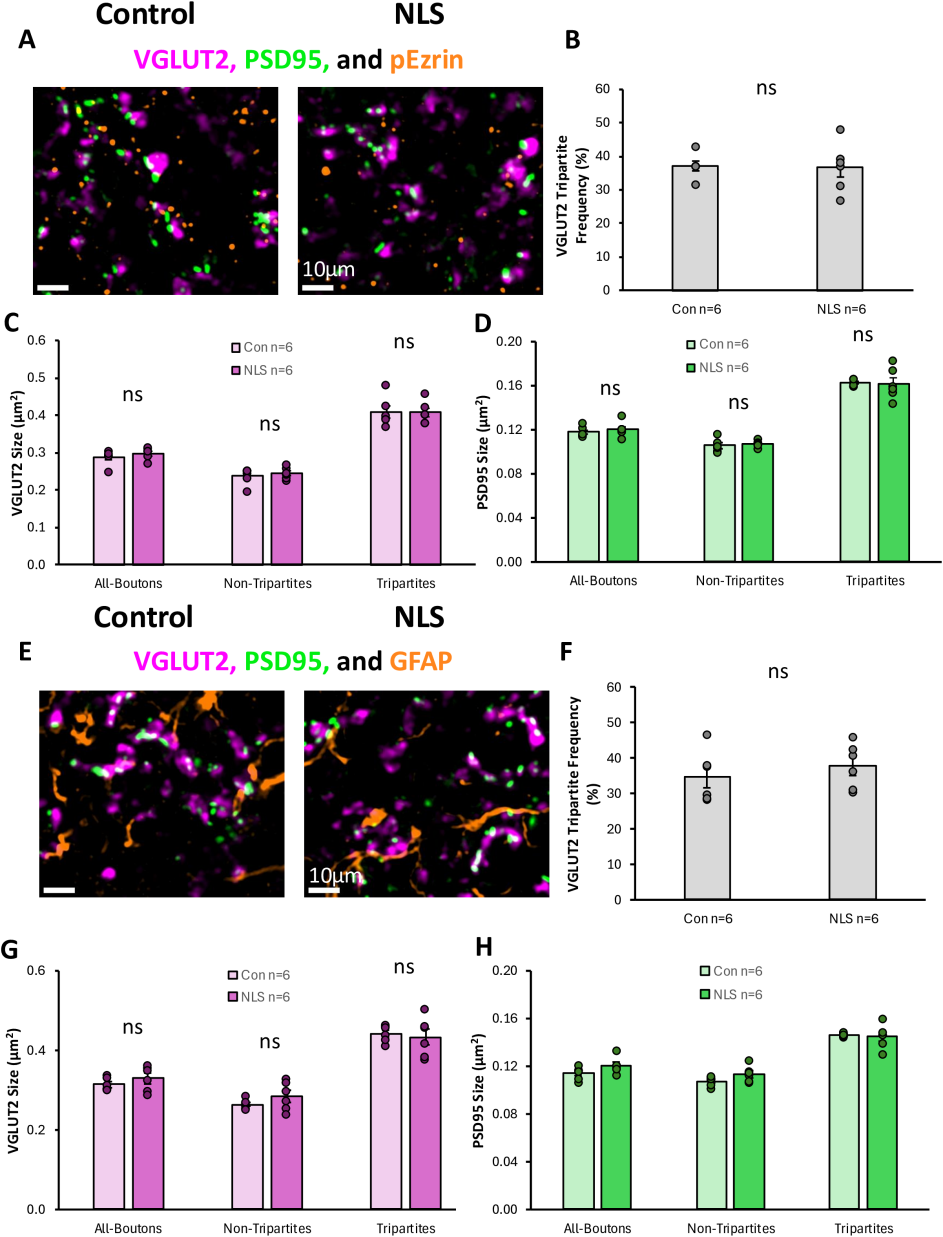
Excitatory VGLUT2‐associated Tripartite Synapses in sALS. (A) Immunohistochemical stain of VGLUT2, PSD95 and pEzrin to identify excitatory tripartite synapses. (B) Bar graph plotting the percentage of VGLUT2 synapses that are tripartite (pEzrin contacted) in control and TDP43ΔNLS (NLS) mice. (C) Bar graph of the size of VGLUT2 boutons associated with all synapses, tripartite and non‐tripartite synapses, in control and NLS mice. (D) Bar graph of the size of PSDs associated with all VGLUT2 synapses, tripartite and non‐tripartite synapses in control and NLS mice. (E) Immunohistochemical stain of VGLUT2, PSD95 and GFAP to identify excitatory tripartite synapses. (F) Bar graph plotting the percentage of VGLUT2 synapses that are tripartite (GFAP contacted) in control and TDP43ΔNLS (NLS) mice. (G) Bar graph of the size of VGLUT2 boutons associated with all synapses, tripartite and non‐tripartite synapses, in control and NLS mice. (H) Bar graph of the size of PSDs associated with all VGLUT2 synapses, tripartite and non‐tripartite synapses in control and NLS mice.

Immunohistochemistry for neurons, synapses and astrocytes was conducted as described previously (Broadhead et al. 2023, 2020, 2022; Sharples et al. 2023). Slides with tissue sections were thawed in a benchtop heater at 37°C for 30 min to aid the adherence of the tissue to the glass slides and reduce tissue loss during subsequent wash steps. Slides were washed three times in PBS. Hydrophobic pens were used to draw rings around each spinal cord tissue on the slides, and the tissue was then incubated in PBS containing 3% Bovine Serum Albumin (BSA; Sigma Aldrich) and 0.2% Triton X100 (Fischer) for 2 h at room temperature to block non‐specific binding and permeabilise the tissue. Primary antibodies were diluted 1:500 in PBS containing 1.5% BSA and 0.1% Triton X100, and samples were incubated with primary antibody solution for two nights at 4°C. Slides were then washed in 1X PBS five times over the course of 1 h. Secondary antibodies were then added to slides, diluted 1:500 in 0.1% Triton X and incubated for 1.5 h. Slides were washed an additional five times in 1X PBS over the course of an hour. If nuclear labelling was performed, DAPI stain was diluted in deionised (DI) water at 1:4000 dilution and applied to the slides for 10 min before being washed in DI water to stop the reaction. Finally, the slides were then dried, and 1.5‐mm‐thick cover slips were applied with Prolong Glass Antifade Mountant (Invitrogen, P36980). A total of 12 mice (control and NLS groups comprised six mice, three male and three female) were used for the behavioural data (Figure 2), TDP‐43 expression (Figure 3), synapse analysis (Figures 4 and 5) and excitatory tripartite synapse analysis (Figures 6 and 7). Analysis of MN number and astrocytic GFAP expression (Figure 3) and cholinergic tripartite synapses was conducted from 11 mice, six control mice and five NLS mice (three male and two female).

Primary and secondary antibodies used are detailed in Table 1.

**TABLE 1:** Details of antibodies used in the study.

Primary antibody species and target	Primary antibody source	Secondary antibody species and fluorophore	Secondary antibody source
Mouse anti‐VGLUT2	Abcam ab79157	Donkey anti‐Mouse Alexa Fluor 405	Invitrogen A31573
Guinea Pig anti‐VGLUT1	Millipore AB5905	Donkey anti‐Guinea Pig Alexa Fluor 647	Jackson Laboratories 706‐605‐148
Rabbit anti‐pTDP43	ProteinTech 22309‐01	Donkey anti‐Rabbit Alexa Fluor 555	Invitrogen, A31570
Camelid anti‐PSD95	Synaptic Systems N3702‐At488‐L	Atto488	Not applicable
Guinea Pig anti‐GLYT2	Synaptic Systems 272‐004	Donkey anti‐Guinea Pig Alexa Fluor 647	Jackson Laboratories 706‐605‐148
Goat anti‐VAChT	Milipore ABN100	Donkey anti‐Goat Alexa Fluor 488	Invitrogen, A11055
Goat anti‐MMP9	Sigma M9570	Donkey anti Goat Alexa Fluor 488	Invitrogen, A11055
Mouse anti‐SMI32	Biolegend 801701	Donkey anti‐mouse Fluor647	Abcam Ab150107
Rabbit anti‐pEzrin	Abcam ab47293	Donkey anti‐Rabbit Alexa Fluor 555	Invitrogen, A31570
Rabbit anti‐GFAP	DAKO Z0334	Donkey anti‐Rabbit Alexa Fluor 555	Invitrogen A31570
Chicken anti‐GFAP	Invitrogen PA110004	Donkey anti‐Chicken Alexa Fluor 594	Sigma SAB4600094

### 2.5 Airyscan Confocal Microscopy

High‐resolution imaging of synapses was performed using the Zeiss LSM800 laser scanning confocal microscope, based on an Axio ‘Observer 7’ microscope, equipped with a 63 × 1.4 NA objective lens, an Airyscan super‐resolution module plus two individual GaAsP PMT detectors. Illumination was provided by 405, 509, 561 and 640 nm laser lines. The pixel size was set to 0.04 μm, resulting in an image size of 78 × 78 μm (2210 × 2210 pixels). Single‐*z* plane images were acquired with bidirectional scanning and 2× averaging, with a pixel scanning time of 2.4 μs. Airyscan processing on images was performed to yield sub‐diffraction limit resolution images of structures, with the Wiener filter kept the same for images within a data set. Images were acquired in the lateral ventral horn, approximately lamina IV, containing pools of MNs.

### 2.6 Andor bc43 Confocal Microscopy

Low‐magnification high‐speed confocal images of mouse lumbar spinal cord tissue were acquired with an Andor bc43 benchtop confocal equipped with 405, 488, 561 and 638 nm lasers for illumination. Z‐stack images were acquired at a 20 × 0.8 NA objective lens. Each multi‐channel acquisition consisted of 11 images acquired across a 2‐μm range in the *Z* axis with a resultant voxel size of 311 × 311 × 181 nm.

### 2.7 Image Analysis

Image analysis was conducted in FIJI (Schindelin et al. 2012). Researchers were blinded to the genotype of animals to prevent bias. Image processing and analysis of synaptic, astrocytic and pTDP‐43 structures was performed using customised macros written in FIJI as used and reported in previous studies (Broadhead et al. 2023, 2020, 2022). Briefly, images undergo processing steps including background subtraction and Gaussian smoothing before being binarized by thresholding. Watershed splitting was applied to segment structures that became merged following thresholding. Small structures consisting of less than eight pixels were filtered out. From the processed images, the number, size and fluorescence intensity of particles was quantified by redirecting the analysis of the binarized images back to the original raw image. Structures were deemed to colocalize when particles overlapped by a minimum of one pixel.

### 2.8 Data Analysis

Data was collated and managed in Microsoft Excel. Statistical analysis was performed in SPSS (IBM, version 28.0). Measurements of synapse or pTDP‐43 cluster density (number per unit area) and size were averaged (median) across all images acquired from at least two tissue sections from individual mice (*n*). Shapiro–Wilks test for normality was performed for measures of synapse size and number, with all data showing normal distributions (SPSS 25.0, IBM). Statistical analysis was performed using a two‐sample *t*‐test. Graphs were generated in Microsoft Excel plotting the mean ± standard error of the mean and figures were constructed in PowerPoint.

## 3 Results

### 3.1 Molecular Hallmarks and Motor Deficits in Mouse Model of sALS

The investigation began by characterising the main hallmarks of sALS in this mouse model: the presence of TDP‐43 mislocalisation and significant motor deficits.

Immunohistochemistry and high‐resolution microscopy were performed on tissue from our experimental cohort of control and TDP43ΔNLS mice (4 weeks post induction), to visualise the expression of pTDP‐43 in cells with DAPI‐labelled nuclei in the ventral horn of the mid‐lumbar spinal cord. pTDP‐43 labelling was predominantly localised to the nucleus, although punctate clusters of TDP‐43 were also seen throughout cell bodies (Figure 2A). To verify the expected mislocalisation of pTDP‐43 in mutant animals, the ratio of pTDP‐43 within DAPI‐labelled nuclei to pTDP‐43 in the surrounding cell body was quantified. In TDP43ΔNLS spinal tissue, cells displayed a reduction or absence of nuclear pTDP‐43 labelling compared to controls (*t*(_10_) = 4.343, *p* = 0.002) (Figure 2B). This confirmed that the experimental cohort of TDP43ΔNLS mice used in this study to model sALS displayed the expected cellular TDP‐43 pathology.

We next confirmed the presence of a significant motor phenotype. The TDP43ΔNLS mice used for this investigation display the same motor phenotypes and phenotypic progression as previously described for this model at 4 weeks post‐induction (Walker et al. 2015; Bak et al. 2023; Djukic et al. 2025). As per previous studies, from 2 to 6 weeks post‐induction (Dox‐withdrawal), mice display progressive hindlimb clasping and tremors, and significantly impaired ability to perform endurance tests such as hanging from a wire or rotarod test (Walker et al. 2015; Bak et al. 2023; Djukic et al. 2025). In our experimental cohort, all TDP43ΔNLS mice showed hindlimb clasping within 5 s of being suspended by their tail, whilst none of the control mice exhibited clasping behaviour during this test (Figure 2C). The endurance time on the grip strength endurance test was significantly reduced in the TDP43ΔNLS mice, whereas all control mice could complete the task for the full 2 min (*t*(_10_) = 71.499, *p* < 0.001) (Figure 2D).

Combined, these data confirm that the TDP43ΔNLS mice used in this study showed the key molecular hallmark (neuronal TDP‐43 mislocalisation) along with significant motor deficits—in accordance with previously published experiments on this model (Walker et al. 2015; Bak et al. 2023; Djukic et al. 2025) and therefore represent an appropriate model for examining the cellular and synaptic hallmarks of sALS.

### 3.2 Cellular Pathology in Mouse Model of sALS

We next asked whether the TDP43ΔNLS mice displayed evidence of cellular pathology in the form of MN degeneration or reactive gliosis. Spinal cord sections were immunolabelled for MMP9 to label a subset of large alpha‐MNs that innervate fast‐twitch muscles (so‐called fast MNs), GFAP to label astrocytes and DAPI to identify cell nuclei. Analysis was conducted to assess changes in the number and size of MMP9‐positive alpha‐MNs in the TDP43ΔNLS mice compared to controls (Figure 3A). There was no difference in the density of MNs in the ventral horn of the lumbar spinal cord, as quantified from the number of MMP9 neurons per 10,000 μm^2^ (*t*(_9_) = 1.351, *p* = 0.201) (Figure 3B). There was, however, a significant reduction in the size of these MNs in the ΔNLS mice, when compared with controls (*t*(_9_) = 5.760, *p* < 0.001) (Figure 3C). This indicates that existing MNs are significantly impacted by the induced TDP‐43 pathology in this model, though the phenotype does not yet lead to significant loss of MNs.

Next, GFAP‐labelling was analysed to determine whether the TDP43ΔNLS mice displayed any signs of reactive gliosis (Figure 3D). A range of parameters was measured, including the density of astrocytes (Figure 3E), fluorescence intensity of GFAP (Figure 3F), the overall area covered by GFAP labelling (Figure 3G) and the branching morphology of the astrocytes (Figure 3H–J). Analysis revealed no changes in the density of the GFAP stained cells (*t*(_9_) = 1.195, *p* = 0.262) (Figure 3E), the fluorescence intensity of GFAP labelling (*t*(_9_) = 0.427, *p* = 0.679) (Figure 3F) or the fraction of area covered by GFAP labelling (*t*(_9_) = 0.107, *p* = 0.917) (Figure 3G) between the TDP43ΔNLS mouse model and non‐transgenic controls. Furthermore, there were no significant differences in the number of branch points (*t*(_9_) = 1.710, *p* = 0.121) (Figure 3I) or the length of branch points (*t*(_9_) = 1.151, *p* = 0.279) of GFAP‐positive astrocytes between the TDP43ΔNLS mice and controls (Figure 3J). This suggests that astrocytes may not be affected by the TDP43ΔNLS mutation present in this model.

In summary, this characterisation reveals that the TDP43ΔNLS mice used in this study displayed TDP‐43 pathology, motor deficits and morphological changes in spinal cord MNs which are consistent with human ALS pathology (Trist et al. 2022) and previous findings using this mouse model (Bak et al. 2023). However, unlike what is seen in human ALS pathology (Vaz et al. 2021), the TDP43ΔNLS mouse model does not show overt astrocytic reactivity.

### 3.3 Differential Vulnerability of Synapses in sALS Model

We next performed a series of immunohistochemical experiments to measure changes in synapse marker expression in TDP43ΔNLS mice. Cholinergic C‐bouton synapses were visualised with vesicular acetylcholine transporter (VAChT) labelling (Figure 4Ai). Glycine transporter 2 (GLYT2) labelling was performed to identify presynaptic boutons of inhibitory synapses (Figure 4Bi). Two different types of excitatory synapses were visualised by labelling for the vesicular glutamate transporter 1 (VGLUT1) (predominantly proprioceptive inputs) (Figure 4Ci) and 2 (VGLUT2) (predominantly synapses from excitatory spinal interneurons) (Figure 4Di). The expression of these four different presynaptic markers was analysed to measure changes in their density (number of synapses per unit area), total synaptic coverage (sum of areas of synaptic labelling) and synapse size (area in μm^2^).

Analysis of VAChT synapses revealed that there was no significant difference in their density, despite an overall trend towards fewer VAChT‐boutons in the TDP43ΔNLS mice compared to controls (*t*(_10_) = 1.848, *p* = 0.094) (Figure 4Aii). However, the total area covered by VAChT labelling was significantly less in TDP43ΔNLS mice compared to controls (*t*(_10_) = 3.805, *p* = 0.003) (Figure 4Aiii). Similarly, the size of VAChT boutons was significantly smaller in TDP43ΔNLS mice compared to controls (*t*(_10_) = 3.932, *p* = 0.003) (Figure 4Aiv).

Analysis of GLYT2‐labelling revealed that there was no significant difference in the number of inhibitory synapses between TDP43ΔNLS mice and controls (*t*(_10_) = 0.104, *p* = 0.919) (Figure 4Bii). Similarly, there was no difference in the total area covered by GLYT2 labelling (*t*(_10_) = 0.265, *p* = 0.796) (Figure 4Biii), or the size of inhibitory presynaptic boutons (*t*(_10_) = 0.146, *p* = 0.444) (Figure 4Biv), in the TDP43ΔNLS mice compared to controls.

Excitatory synapses were visualised using both VGLUT1 (Figure 4Ci) and VGLUT2 (Figure 4Di) labelling. We found a lower density of VGLUT1 synapses in the TDP43ΔNLS mice compared to controls (*t*(_10_) = 2.278, *p* = 0.046) (Figure 4Cii). Meanwhile, there was no difference between TDP43ΔNLS mice and controls in the total area of VGLUT1 labelling (*t*(_10_) = 1.793, *p* = 0.103) (Figure 4Ciii) or the average size of VGLUT1 boutons (*t*(_10_) = 0.364, *p* = 0.723) (Figure 4Civ). In contrast, there was no difference in VGLUT2 synaptic density (*t*(_10_) = 1.259, *p* = 0.237) (Figure 4Dii), total area covered by VGLUT2 labelling (*t*(_10_) = 2.059, *p* = 0.066) (Figure 4Diii), or the size of VGLUT2 boutons (*t*(_10_) = 0.417, *p* = 0.685) (Figure 4Div) in TDP43ΔNLS mice compared to controls.

From analysis of these individual synaptic markers in areas around MNs in the mouse spinal cord, significant and selective synaptic pathology is observed. Our results highlight that cholinergic C‐boutons and VGLUT1‐associated excitatory synapses are most significantly impacted in the TDP43ΔNLS model of sALS, whilst inhibitory synapses (GLYT2) and the majority of excitatory synapses (VGLUT2) are not significantly affected.

### 3.4 TDP‐43 Expression in Different Synapse Subtypes

Following the assessment of cholinergic, inhibitory and excitatory synapses (Figure 4), and the previous observation of cytoplasmic mis‐localisation of TDP‐43 (Figure 3A,B), the presence of pTDP‐43 pathology within these synapses was examined.

We found a significantly lower frequency of VAChT boutons that contained pTDP‐43 (approximately 74%) in TDP43ΔNLS mice compared to non‐transgenic controls (approximately 80%) (*t*(_10_) = 2.405, *p* = 0.037) (Figure 5Aii). Additionally, there were significantly fewer pTDP‐43 clusters per VAChT bouton in the TDP43ΔNLS model (approximately two clusters per bouton) compared to controls (approximately three clusters per bouton) (*t*(_10_) = 3.946, *p* = 0.003) (Figure 5Aiii). In examining the size of the pTDP‐43 clusters localised within the VAChT boutons, there was no significant difference (*t*(_10_) = 2.020, *p* = 0.071) (Figure 5Aiv).

No significant differences were observed in the frequency of GLYT2 synapses containing pTDP‐43 (*t*(_10_) = 0.318, *p* = 0.757) (Figure 5Bii), the number of pTDP‐43 puncta colocalized per GLYT2 synapse (*t*(_10_) = 0.280, *p* = 0.785) (Figure 5Biii) or the average size of the pTDP‐43 puncta associated with GLYT2 synapses (*t*(_10_) = 0.651, *p* = 0.529) (Figure 5Biv), when comparing the TDP43ΔNLS mouse model and non‐transgenic controls.

Similarly, no differences were seen between groups when assessing the frequency of pTDP‐43 colocalized with VGLUT1 synapses (*t*(_10_) = 0.147, *p* = 0.886) (Figure 5Cii), the number of pTDP‐43 puncta per VGLUT1 synapse (*t*(_10_) = 0.320, *p* = 0.756) (Figure 5Ciii) or the size of pTDP‐43 associated with VGLUT1 synapses (*t*(_10_) = 0.966, *p* = 0.357) (Figure 5Civ). Finally, there was also no difference in the frequency of VGLUT2 synapses associated with pTDP‐43 (*t*(_10_) = 0.398, *p* = 0.699) (Figure 5Dii), the number of pTDP‐43 puncta colocalized per VGLUT2 synapse (*t*(_10_) = 0.448, *p* = 0.664) (Figure 5Diii) or the size of the pTDP‐43 puncta associated with VGLUT2 synapses (*t*(_10_) = 1.225, *p* = 0.249) (Figure 5Div).

Our findings show synapse subtype‐specific changes in TDP‐43 expression. We observed reduced association of TDP‐43 with C‐boutons; however, the other excitatory and inhibitory synapse populations showed no evidence of changes in their content of TDP‐43.

### 3.5 Tripartite Synapses in sALS Model

We have previously shown that excitatory tripartite synapses are selectively vulnerable to degeneration in the commonly used SOD1^G93a^ model of fALS, and in human cases based on analysis of post‐mortem tissue from ALS patients with C9ORF72 mutations (Broadhead et al. 2022). We therefore hypothesised that tripartite synapses may also represent selectively vulnerable synapse subtypes in the TDP43ΔNLS mouse model of sALS (Broadhead et al. 2022).

Excitatory synapses were examined by labelling for presynaptic VGLUT1 and VGLUT2 and the postsynaptic marker PSD95. For these assessments, synapses were defined as presynaptic boutons partially colocalised with PSD95. Synapses were assigned as tripartite or non‐tripartite based on partial colocalization with the perisynaptic astrocytic process (PAP) marker, phosphorylated‐Ezrin (p‐Ezrin).

Approximately 75% of VGLUT1‐PSD95 synapses in the control animals were classified as tripartite synapses based on their association with p‐Ezrin puncta (Figure 6A). There was no significant difference in the percentage of VGLUT1‐synapses that were tripartite between TDP43ΔNLS and control mice (*t*(_10_) = 0.243, *p* = 0.813) (Figure 6B). The size of VGLUT1 boutons from all bona fide synapses was no different between TDP43ΔNLS and control mice (*t*(_10_) = 0.571, *p* = 0.580) (Figure 6C). Similarly, there was no difference in VGLUT1 bouton size between TDP43ΔNLS and control mice whether they were part of tripartite synapses (*t*(_10_) = 0.511, *p* = 0.620) or non‐tripartite synapses (*t*(_10_) = 1.230, *p* = 0.247) (Figure 6C). Whilst there was no change in VGLUT1 presynaptic bouton morphology, the size of the opposed PSD95 puncta (the PSDs) was significantly smaller in TDP43ΔNLS mice compared to controls (*t*(_10_) = 4.014, *p* = 0.00246). When synapses were analysed based on their association with p‐Ezrin PAPs, the PSDs that were part of non‐tripartite synapses were significantly smaller in TDP43ΔNLS mice compared to controls (*t*(_10_) = 2.946, *p* = 0.015). In contrast, PSDs that were part of tripartite synapses were not significantly smaller in TDP43ΔNLS mice (~0.19 μm^2^) compared to controls (~0.17 μm^2^) (Figure 6D).

For further validation, we repeated this experiment with an alternative astrocytic marker, GFAP (Figure 6E). There was also no difference in the percentage of VGLUT1 synapses contacted by pEzrin (approximately 50%) between control and TDP43ΔNLS mice (Figure 6F). Similarly, there was no difference in the size of VGLUT1 presynaptic boutons, whether they were tripartite synapses (*t*(10) = 1.77, *p* = 0.108) or non‐tripartite synapses (*t*(10) = 1.14, *p* = 0.282) (Figure 6G). We observed that the PSDs associated with all VGLUT1 synapses were significantly smaller in TDP43ΔNLS mice compared to controls (*t*(_10_) = 3.666, *p* = 0.004), whether they were associated with tripartite synapses (*t*(_10_) = 2.413, *p* = 0.037) or non‐tripartite synapses (*t*(_10_) = 3.047, *p* = 0.0123) (Figure 6H). Taken together, our data indicate that VGLUT1 synapses display postsynaptic alterations that are independent of astrocytic interactions.

Analysis of VGLUT2‐associated synapses and p‐Ezrin‐associated tripartite synapses revealed that approximately 38% of all VGLUT2‐associated excitatory synapses were classified as tripartite synapses based on their association with p‐Ezrin puncta (Figure 7A). There was no significant difference in the percentage of VGLUT2‐synapses that were tripartite between TDP43ΔNLS and control mice (*t*(_10_) = 0.014, *p* = 0.989) (Figure 7B). The size of VGLUT2 presynaptic boutons was not significantly different between TDP43ΔNLS and control mice (*t*(_10_) = 1.036, *p* = 0.325) (Figure 7C). There was also no difference in VGLUT2 bouton size between TDP43ΔNLS and control mice whether they were part of tripartite synapses (*t*(_10_) = 0.108, *p* = 0.916) or non‐tripartite synapses (*t*(_10_) = 0.648, *p* = 0.532) (Figure 7C). When we analysed the PSDs of VGLUT2‐associated synapses, there was also no significant difference in their size between TDP43ΔNLS mice and controls, whether from all VGLUT2‐associated synapses (*t*(_10_) = 0.708, *p* = 0.495), tripartite synapses (*t*(_10_) = 0.117, *p* = 0.910) or non‐tripartite synapses (*t*(_10_) = 0.686, *p* = 0.508) (Figure 7D).

Similarly, we observed no change in the percentage of VGLUT2 synapses that were contacted by GFAP‐positive structures between TDP43ΔNLS mice and controls (Figure 7E,F). There was also no change in the size of VGLUT2 presynaptic boutons, whether they were part of GFAP‐associated tripartite synapses or not (Figure 7G). VGLUT2‐associated PSDs were not significantly different between TDP43ΔNLS mice and controls, whether or not they were associated with tripartite synapses (*t*(_10_) = 0.378, *p* = 0.714) or non‐tripartite synapses (*t*(_10_) = 1.936, *p* = 0.082) (Figure 7H).

Overall, our findings reveal selective changes in postsynaptic structures of VGLUT1‐associated synapses, but not VGLUT2‐associated synapses. Our findings also show that, contrary to our hypothesis, there is no selective vulnerability of excitatory tripartite synapses in the TDP43ΔNLS mice.

**FIGURE 8:**
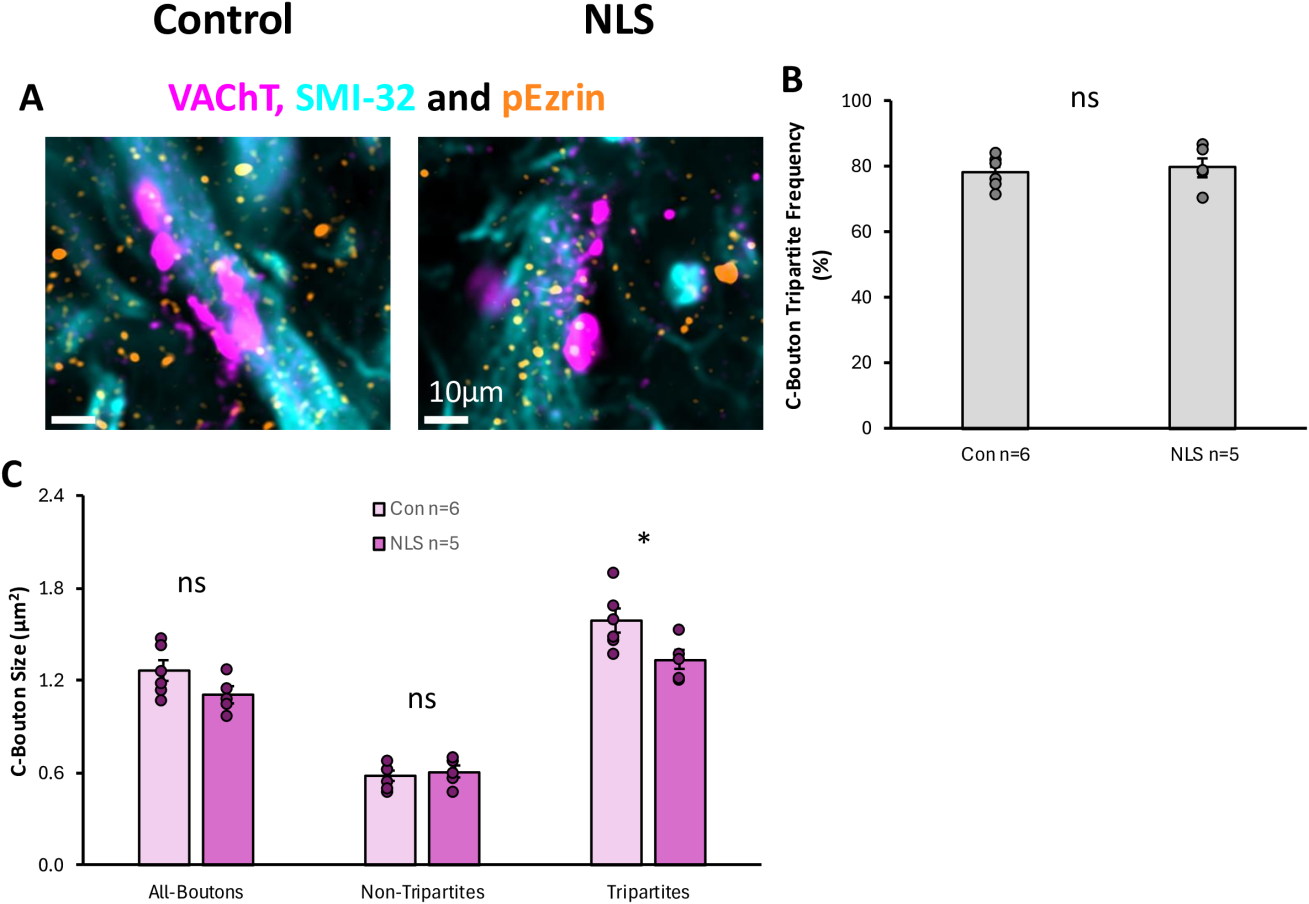
Cholinergic VAChT C‐boutons and Tripartite Synapses in sALS. (A) Immunohistochemical stain of VAChT, SMI‐32 and pEzrin to identify C‐boutons and identify whether they are tripartite synapses or not. (B) Bar graph plotting the percentage of C‐boutons that are tripartite (pEzrin contacted) in control and TDP43ΔNLS (NLS) mice. (C) Bar graph of the size of VAChT C‐boutons associated with all synapses, tripartite and non‐tripartite synapses.

We next investigated whether astrocytic contacts with cholinergic C‐boutons were altered in the TDP43ΔNLS model of sALS. C‐boutons were identified as VAChT‐positive puncta opposed to SMI‐32 labelling, which broadly labelled the soma and processes of MNs. VAChT‐boutons that were partially colocalised with p‐Ezrin puncta were classified as tripartite synapses (Figure 8A).

Approximately 80% of all C‐boutons were associated with p‐Ezrin PAPs (Figure 8B). There was no difference in the percentage of tripartite C‐bouton synapses between control and TDP43ΔNLS mice (*t*(9) = 0.469, *p* = 0.650). Interestingly, however, C‐boutons associated with p‐Ezrin PAPs were significantly smaller in TDP43ΔNLS mice compared to controls (*t*(_9_) = 2.521, *p* = 0.033). In contrast, there was no significant difference in the size of C‐boutons that were not contacted by p‐Ezrin PAPs (*t*(_9_) = 0.596, *p* = 0.566) (Figure 8C). These data may indicate selective morphological changes in tripartite C‐bouton synapses.

## 4 Discussion

### 4.1 TDP43ΔNLS Mouse Model for sALS

This study makes use of genetically inducible TDP‐43 mislocalisation in neurons to drive molecular pathology and motor behavioural changes resembling ALS in order to identify which types of synapses are most significantly vulnerable to degeneration in the disease. Many TDP‐43 mouse models have been generated, which have enabled studies of TDP‐43 over‐expression and TDP‐43 loss of function. These studies have revealed a spectrum of effects on mice including changes in weight (both increased and reduced) and cognitive and motor dysfunction that can occur with or without classical TDP‐43 mislocalisation from the nucleus to the soma (Igaz et al. 2011; Xu et al. 2011, 2010; Wils et al. 2010; Swarup et al. 2011; Watkins et al. 2021).

Igaz et al. (2011) originally used the Camk2a promotor to drive disruption of the NLS sequence of TDP‐43 predominantly in upper MNs, sparing lower spinal MNs and thus resembling a mouse model of peripheral lateral sclerosis (PLS) (Igaz et al. 2011). Walker et al. (2015) then used the NEFH promotor to induce TDP‐43 mislocalisation in both the brain and spinal cord, thus more closely resembling a model of ALS (Walker et al. 2015).

The TDP43ΔNLS mice with the NEFH promotor used in this study and others' (Walker et al. 2015; Bak et al. 2023; Djukic et al. 2025) show a rapid inducible ALS‐like pathology accompanied by severe motor deficits. By 2 weeks post‐induction, more than 75% of mice show hindlimb clasping, almost 50% of mice display a tremor, and the mice first display reductions in grip strength and endurance, as measured by wirehang tests and rotarod tests (Walker et al. 2015). By 4 weeks post‐induction, all mice display hindlimb clasping and tremors, a significant loss in body mass and more prevalent motor deterioration—exemplified clearly by wirehanging endurance test scores whereby symptomatic mice only last 10–20 s before falling, whilst control animals can last at least 2 min (Walker et al. 2015; Djukic et al. 2025). After 6 weeks post‐induction, TDP43ΔNLS mice show approximately 20% weight loss and mortality begins to increase (average survival of ~10 weeks post‐induction) (Walker et al. 2015).

In comparison, the commonly used fALS model, SOD1^G93a^ (SOD1), displays early‐stage phenotypes of hindlimb clasping and tremors when raised by the tail at approximately 70–90 days of age, and by approximately 120 days animals show rotarod and wire‐hang endurance scores comparable to that of a 4‐week induced TDP43ΔNLS mouse (Pfohl et al. 2015; Hatzipetros et al. 2015; Oliván et al. 2014). We therefore reason that the 4‐week induced cohort of TDP43ΔNLS mice used in our study is comparable to the late‐stage 120‐day‐old SOD1 mouse model and is thus appropriate for identifying which types of synapses are most significantly vulnerable to degeneration.

### 4.2 C‐Boutons in sALS Model

Our research demonstrated significant changes in cholinergic C‐boutons in the TDP‐43ΔNLS mice. C‐boutons, which arise from a population of Pitx2‐expressing cholinergic interneurons near the central canal of the spinal cord, are distinctively large presynaptic boutons that are associated with a complex post‐synaptic organisation including subsurface cisternae (Nagy et al. 1993; Witts et al. 2014; Zagoraiou et al. 2009; Deardorff et al. 2014; Rozani et al. 2019). C‐boutons modulate the excitability of MNs through activation of muscarinic (m2) receptors and regulation of Kv2.1 channels that lead to reduced spike half‐width (Miles et al. 2007; Nascimento et al. 2020, 2019).

Our observation of overall degeneration of C‐boutons replicates prior work in the TDP‐43ΔNLS model showing a loss of C‐bouton volume and a selective decrease in the number of C‐boutons onto fast‐type MNs innervating the gastrocnemius muscle (Bak et al. 2023). The reduced number of C‐boutons in the TDP‐43ΔNLS model is partly accounted for by the reduced size of MNs. When soma size is taken into consideration, gastrocnemius MNs in fact display an increased density of C‐boutons contacting the soma (Bak et al. 2023). We similarly observed reduced size of MMP9‐labelled fast‐type MNs, which account for approximately half the total number of MNs in mid‐lumbar ventral horn lateral motor pools (Sharples et al. 2023; Kaplan et al. 2014).

Comparably, human post‐mortem studies display significantly reduced numbers of VAChT‐positive boutons around the surviving MNs in tissue from sALS patients, despite unchanged levels of immunoreactivity for the more ubiquitous presynaptic marker, synaptophysin (Nagao et al. 1998)—broadly supporting the concept of highly selective synaptic vulnerability.

C‐boutons are also smaller in size in the TDP43ΔNLS model. This appears in contrast to the SOD1 model which displays increased C‐bouton sizes from early pre‐symptomatic stages, which likely acts as a compensatory mechanism for spinal networks (Herron and Miles 2012; Wells et al. 2021; Landoni et al. 2019). One explanation for this difference could be an impact of the SOD1 mutation on the formation of synaptic circuits during development, which is not possible in the TDP43ΔNLS model due to its rapid disease progression in adulthood. Alternatively, this could be a specific effect of the TDP‐43 pathology observed in this model, highlighting the importance of utilising models other than the traditional SOD1 model.

Whilst defining the fate of C boutons helps extend our understanding of ALS disease mechanisms, C‐boutons also represent potential therapeutic targets due to their modulation of MN function. Experimental silencing of C‐boutons has been shown to improve muscle innervation, and combined with task‐dependent muscle training, may improve behavioural capabilities of SOD1 animals (Wells et al. 2021), although, our results suggest that this may not necessarily have the same effect in models exhibiting TDP‐43 pathology.

### 4.3 Inhibitory Synapses in sALS Model

There is considerable interest in the contribution of different spinal inhibitory interneuron subtypes to the early stages of ALS (Lorenc et al. 2024), though there is mixed evidence from human ALS cases. Patients with Frontotemporal Dementia (FTD) were found to have reduced cortical GABA (Murley et al. 2021), whilst GAD mRNA levels were found to be increased in cortical regions of ALS patients (Petri et al. 2006). Another study reported an upregulation of GABAergic system‐related genes such as GAD1, GABRA1, GABRD and GABRB2, although in some cases these were not associated with protein level changes (Andrés‐Benito et al. 2017).

From analysis of the ubiquitous inhibitory synapse marker, GLYT2, we observed no significant change in inhibitory synapses in the TDP43ΔNLS model. Recently, we hypothesised that early developmental changes in excitatory and inhibitory synapses in ALS could pre‐dispose spinal motor circuits to an excitatory/inhibitory imbalance, leading to hyperexcitability and excitotoxicity (Foerster et al. 2013). Our findings from the SOD1 and C9orf72 mouse models showed there was no such early‐stage imbalance in the number of excitatory and inhibitory synapses in spinal neurons both in vitro and in vivo in the first 30 postnatal days (Bonthron et al. 2024).

However, given the molecular and anatomical diversity of inhibitory synapses from different microcircuits, it is possible that analysis using a pan‐inhibitory synapse marker such as GLYT2 may occlude subtype‐specific changes. Indeed, other studies in mouse models have demonstrated a reduced number of inhibitory synapses in the brain and spinal cord (Petri et al. 2006; Malessa et al. 1991; Ramírez‐Jarquín et al. 2014). Studies in the SOD1 mouse model at slightly later, albeit early or presymptomatic, stages (30–60 days) demonstrate both functional and structural changes in inhibitory synaptic input to MNs (Nascimento et al. 2024; Allodi et al. 2021; Mora et al. 2024). Spatial transcriptomics has shown a significant loss of V1‐inhibitory interneurons that precedes the loss of MNs (Montañana‐Rosell et al. 2024), whilst genetic stabilisation of a vulnerable V1 inhibitory interneuron subpopulation ameliorates the motor phenotype in the SOD1 model (Mora et al. 2024). Functional analysis of recurrent inhibition to MNs found that it was significantly reduced in juvenile SOD1 animals, which correlated with reduced density of glycine receptor clusters (Nascimento et al. 2024). Interestingly, whilst these changes were observed in young juvenile animals (14–25 days old), in adulthood (40–60 days old), they were comparable to healthy controls suggesting compensatory mechanisms. It is therefore possible that the sporadic TDP43ΔNLS mouse model used in our study, could have shown early adaptive changes in distinct inhibitory circuits to MNs in the first weeks following induction that are then compensated for by 3–4 weeks post‐induction.

### 4.4 Excitatory Synapse Changes in sALS Model

Our results highlight a selective vulnerability of VGLUT1‐associated excitatory synapses, whilst VGLUT2‐associated synapses were unchanged in their size and number. Within the spinal cord, the majority of VGLUT2 boutons originate from local spinal neurons, with a smaller number arising from nociceptive inputs (Todd et al. 2003; Lagerström et al. 2010) and descending inputs from the rubrospinal and vestibulospinal tracts (Du Beau et al. 2012). VGLUT1 primarily labels Ia afferents from dorsal root ganglia sensory neurons to provide excitatory proprioceptive feedback to large alpha MNs that are most vulnerable to degeneration in ALS (Todd et al. 2003; Kaplan et al. 2014; Comley et al. 2015; Nijssen et al. 2017; Lalancette‐Hebert et al. 2016; Ni et al. 2014).

Our findings demonstrate a loss of VGLUT1 synapses and a reduced size of PSD‐95 clusters associated with VGLUT1‐boutons. In our previous study, which examined synaptic pTDP‐43 clusters in mouse spinal cord tissue, we similarly demonstrated that VGLUT1‐associated synapses are more substantially affected in the SOD1 model compared to VGLUT2‐associated synapses (Broadhead et al. 2023). Other studies have shown functional and structural changes in VGLUT1‐positive Ia afferents to MNs, suggesting a time course of early‐stage increases in the strength of proprioceptive inputs, followed by reduced strength, reduced postsynaptic clustering and finally a loss of presynaptic VGLUT1 inputs to MNs and impaired proprioception in patients (Broadhead et al. 2023; Bączyk et al. 2020; Andrés‐Benito et al. 2017; Vaughan et al. 2018, 2015; Simmatis et al. 2019). Spinal muscular atrophy similarly shows early functional and structural impairment in Ia afferent connections to MNs (Mentis et al. 2011; Fletcher et al. 2017). Recent attempts using trans‐spinal direct current stimulation have been successful in restoring Ia afferent structure and function in SOD1 animals, although there was no change in the disease burden in the animals, as determined by the expression of phosphorylated pCREB (Jankowiak et al. 2024).

The concept of a conserved VGLUT1‐associated synaptic phenotype leading to comparable motor deficits in two mouse models (i.e., SOD1 and TDP43ΔNLS) with fundamentally distinct genetic and pathological mechanisms, is of particular interest for understanding the molecular mechanisms of ALS. This may implicate other shared downstream targets of both SOD1 and TDP‐43. Indeed, there is conflicting evidence on the role of TDP‐43 in ALS synaptopathy. TDP‐43 is present at synapses (Broadhead et al. 2023), it is implicated in the translation of synaptic proteins (Tollervey et al. 2011; Godena et al. 2011), and mutations in the gene encoding TDP‐43 (TARDBP) lead to altered dendritic arborisation, spine formation and synapse numbers in cortical neurons and corticospinal circuits (Fogarty et al. 2016; Reale et al. 2023; Dyer et al. 2021). Synaptic analysis from human ALS post‐mortem brain tissue shows a correlation between patient TDP‐43 pathology and the degree of synapse loss (Henstridge et al. 2018). However, in the spinal cord of ALS patients, synapse loss does not correlate with the number of neurons showing TDP‐43 pathology (Aousji et al. 2023).

### 4.5 No Astrocyte Reactivity to Neuronal TDP‐43 Pathology

In this model the TDP‐43 is exclusively driven in neurons using a NEFH promoter, leading to TDP‐43 mislocalisation and early degeneration in spinal neurons. This allows us to determine the neuron‐autonomous effects of the pathology. This is important as there is considerable evidence of non‐cell‐autonomous mechanisms either driving or exacerbating pathology in ALS (Zhao et al. 2020; Marchetto et al. 2008; Tong et al. 2013). Early histological analysis demonstrated evidence of gliosis in human ALS patient post‐mortem spinal cords in both the ventral horn and near corticospinal tracts (Schiffer et al. 1996). The use of human ALS patient‐derived induced pluripotent stem cells differentiated into astrocytes has demonstrated evidence of their toxicity to cultured neurons, suggesting a primary role in the disease mechanism (Zhao et al. 2020; Haidet‐Phillips 2011; Di Giorgio et al. 2008; Devlin et al. 2015). Molecular studies implicate changes in neural‐glial signalling mechanisms in the spinal cord of the SOD1 mouse model (MacLean et al. 2022). Histologically a selective loss of tripartite synapses across ventral and intermediate laminae of the lumbar spinal cord is observed in the SOD1 mouse model and in post‐mortem tissue from ALS patients (Broadhead et al. 2022). Spinal cord tripartite synapses are structurally and molecularly enriched in PSD95, which may in part be driven by astrocyte activity (Broadhead et al. 2020). Tripartite synapses harbour the molecular machinery for a bi‐directional neural‐glial signalling mechanism whereby astrocytes provide modulatory feedback inhibition of spinal motor networks that may act to prevent hyperexcitability (Broadhead and Miles 2021). Many individual components of this neural‐glial signalling pathway, such as mGluR5 signalling and purinergic signalling have been independently verified as dysregulated in ALS models and patients (Cieślak et al. 2019; Ng et al. 2015; Ferré et al. 2002; Bonifacino et al. 2019; Aronica et al. 2001).

Given this body of information, we hypothesised that tripartite synaptopathy is a conserved hallmark of ALS. However, our findings from this TDP43ΔNLS model suggest that there is no evidence of astrocyte pathology or selective excitatory tripartite synapse loss. For example, the overall fraction of either VGLUT1‐associated or VGLUT2‐associated excitatory tripartite synapses was unchanged, and structural changes in PSDs were independent of astrocytic contacts. Although C‐boutons showed a degree of selective structural changes when associated with p‐Ezrin, there was no overall change in the fraction of C‐boutons contacted by p‐Ezrin. Indeed, unlike previous observations from the SOD1 model, there were no changes in the number of p‐Ezrin PAPs in the TDP43ΔNLS spinal cord.

These data support there being no such astrocyte pathology in this model. Therefore, whilst there is interest in the non‐cell autonomous mechanisms of ALS, such as astrocytic or microglial cell pathology, some of the synaptic hallmarks of the disease (such as structural changes and synapse loss) may in fact be driven by neuronal mechanisms.

Understanding the differential contribution of TDP‐43 pathology in neuronal and glial cell populations, separately and combined, is necessary to dissect the cellular mechanisms of ALS. Future work driving the TDP43ΔNLS genes selectively in glia or other cell types will allow us to further address this.

## 5 Conclusion

Whilst there is evidence that synapses are structurally and functionally altered in the early stages of ALS, what is lacking is a comprehensive understanding of which types of synapses are vulnerable. Our investigation begins to tackle this by examining a range of synapse subtypes in the spinal cord and contributing to evidence of selective synapse vulnerability in a model of sALS. We also observe hallmarks of synaptopathy that are seemingly conserved across models of ALS, indicating different molecular mechanisms underlying ALS may converge on particular synapses and circuits.

## Author Contributions

**Ani Ayvazian‐Hancock:** conceptualization, data curation, investigation, methodology, writing – original draft, writing – review and editing. **Emma Butler:** formal analysis, investigation, methodology, writing – original draft. **Claire F. Meehan:** formal analysis, funding acquisition, investigation. **Gareth B. Miles:** supervision, writing – review and editing. **Matthew J. Broadhead:** conceptualization, data curation, funding acquisition, investigation, methodology, project administration, supervision, writing – original draft, writing – review and editing.

## Conflicts of Interest

The authors declare no conflicts of interest.

## Data Availability

The research data underpinning this publication can be accessed at https://doi.org/10.17630/1a106a80‐836f‐457f‐8660‐307d817dabb2. The original raw files are available upon request to the corresponding author.
